# Supplementary material for: Molecular Networking-Guided Annotation of Flavonoid Glycosides from Quercus mongolica Bee Pollen
Source: Int J Mol Sci. 2025 Aug 17;26(16):7930. doi: 10.3390/ijms26167930 (PMC12386960; doi:10.3390/ijms26167930)
Supplement: Supplementary file 1 [file ijms-26-07930-s001.zip › ijms-3788953-supplementary.pdf]

Molecular Networking-Guided Annotation of Flavonoid Glycosides from *Quercus mongolica* Bee Pollen

Yerim Joo[1,2], Eunbeen Shin[1,2], Hyunwoo Kim[3], Mi Kyeong Lee[4,*] and Seon Beom Kim[1,2,5,*]

[1]Department of Food Science and Technology, Pusan National University, Miryang 50463, Republic of Korea

[2] Institute for Future Earth, Pusan National University, Busan 46421, Republic of Korea

[3]College of Pharmacy and Integrated Research Institute for Drug Development, Dongguk University-Seoul, Goyang 10326, Republic of Korea

[4]College of Pharmacy, Chungbuk National University, Cheongju 28160, Republic of Korea

[5]Food Tech Innovation Center, Life and Industry Convergence Research Institute, Pusan National University, Miryang 50463, Republic of Korea

Supporting Information

■ **Table of Contents**

Table S1. ^{1}H and ^{13}C NMR spectral data for compounds **28** and **30** (^{1}H and ^{13}C chemical shifts were recorded at 400 MHz and 100 MHz in CD_3OD).

Figure S1. MS^2 spectrum of quercetin 3-*O*-sambubioside (**A1**, compound **6**; Rt = 4.17 min; $[M-H]^-$ *m/z* 595.132).

Figure S2. MS^2 spectrum of isorhamnetin 3-*O*-*β*-D-xylopyranosyl(1→6)-*β*-D-glucopyranoside

(**A3**, compound **28**; Rt = 5.47 min; $[M-H]^-$ *m/z* 609.148).

Figure S3. ^{1}H NMR spectrum (400 MHz, CD_3OD) of compound **28**.

Figure S4. ^{13}C NMR spectrum (100 MHz, CD_3OD) of compound **28**.

Figure S5. ^{1}H-^{1}H COSY spectrum (400 MHz, CD_3OD) of compound **28**.

Figure S6. ^{1}H-^{13}C edited HSQC spectrum (100 MHz, CD_3OD) of compound **28**.

Figure S7. ^{1}H-^{13}C HMBC spectrum (100 MHz, CD_3OD) of compound **28**.

Figure S8. MS^2 spectrum of isorhamnetin 3-*O*-neohesperidoside (**A2**, compound **30**; Rt = 4.75 min; $[M-H]^-$ *m/z* 623.165).

Figure S9. ^{1}H NMR spectrum (400 MHz, CD_3OD) of compound **30**.

Figure S10. ^{13}C NMR spectrum (100 MHz, CD_3OD) of compound **30**.

Figure S11. ^{1}H-^{1}H COSY spectrum (400 MHz, CD_3OD) of compound **30**.

Figure S12. ^{1}H-^{13}C edited HSQC spectrum (100 MHz, CD_3OD) of compound **30**.

Figure S13. ^{1}H-^{13}C HMBC spectrum (100 MHz, CD_3OD) of compound **30**.

Table S1.^{1}H and ^{13}C NMR spectral data for compounds **28** and **30** (^{1}H and ^{13}C chemical shifts were recorded at 400 MHz and 100 MHz in CD_3OD)

Position	Compound **28**		Compound **30**	
	δ_H, multi (*J* in Hz)	δ_C, type	δ_H, multi (*J* in Hz)	δ_C, type
**2**	-	158.59, C	-	158.47, C
**3**	-	135.40, C	-	134.30, C
**4**	-	179.20, C	-	179.25, C
**5**	-	162.99, C	-	163.18, C
**6**	6.19, d (1.95)	100.33, CH	6.17,d (1.95)	99.98, CH
**7**	-	167.25, C	-	166.60, C
**8**	6.4, d (1.95)	95.14, CH	6.37, d (1.95)	94.74, CH
**9**	-	158.32, C	-	158.14, C
**10**	-	105.43, C	-	105.77, C
**1'**	-	123.00, C	-	123.42, C
**2'**	8.02, d (1.9)	114.48, CH	7.97, d (1.95)	114.51, CH
**3'**	-	148.42, C	-	148.39, C
**4'**	-	150.92, C	-	150.62, C
**5'**	6.91, d (8.4)	116.09, CH	6.91, d(8.4)	115.99, CH
**6'**	7.65, dd (1.9. 8.4)	123.76, CH	7.55, dd (1.95. 8.4)	123.49, CH
**3'-OCH$_3$**	3.95, s	56.91, CH_3	3.97, s	56.93, CH_3
**1''**	5.33, d (7.4)	103.95, CH	5.89, d (7.4)	100.19, CH
**2''**	3.49, m	74.78, CH	3.63, m	80.32, CH
**3''**	3.45, m	77.81, CH	3.58, m	78.88, CH
**4''**	3.36, m	71.26, CH	3.28, m	71.85, CH
**5''**	3.39, m	78.00, CH	3.25, m	78.44, CH
**6''**	3.93, dd(1.4, 11.8)<br>3.64, dd (5.3, 11.8)	69.33, CH_2	3.77, m<br>3.55, m	62.49, CH_2
**1'''**	4.09, d (7.2)	105.05, CH	5.18, d (1.2)	102.77, CH
**2'''**	3.05, dd (7.2, 8.9)	75.95, CH	3.99, m	72.33, CH
**3'''**	3.10, m	77.55, CH	3.75, m	72.39, CH
**4'''**	3.33, m	71.02, CH	3.30, m	73.97, CH
**5'''**	3.62, dd (5.3, 11.4)<br>2.90, dd(10.3, 11.4)	66.59, CH_2	4.00, m	69.88, CH
**6'''**	-	-	0.88, d (6.2)	17.38, CH_3

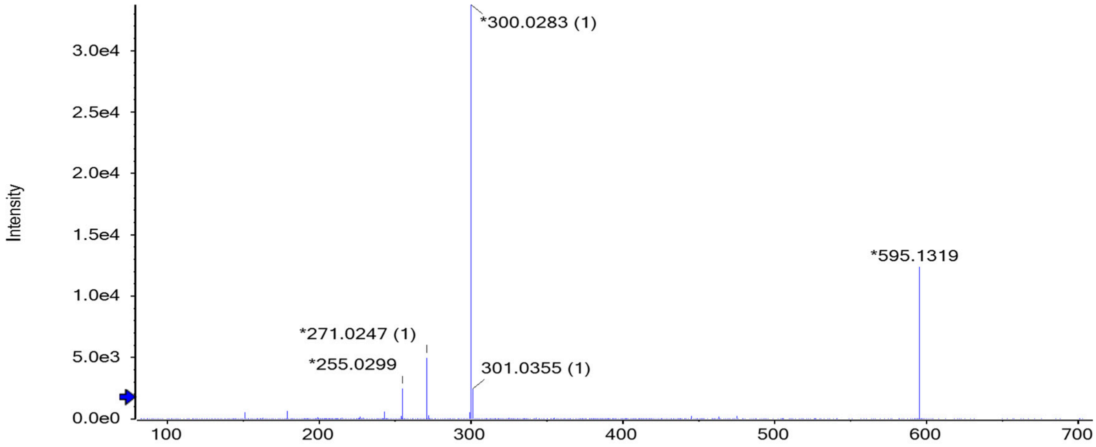

Figure S1. MS[2] spectrum of quercetin 3-*O*-sambubioside (**A1**, compound **6**; Rt = 4.17 min; [M−H]$^-$ *m/z* 595.132)

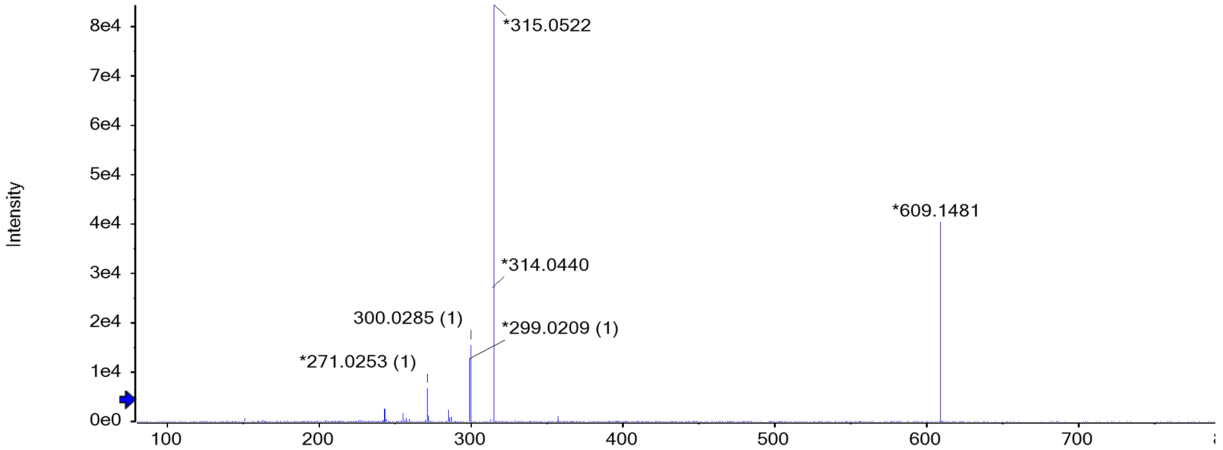

Figure S2. MS[2] spectrum of isorhamnetin 3-*O*-*β*-D-xylopyranosyl(1→6)-*β*-D-glucopyranoside (**A3**, compound **28**; Rt = 5.47 min; [M−H]$^-$ *m/z* 609.148)

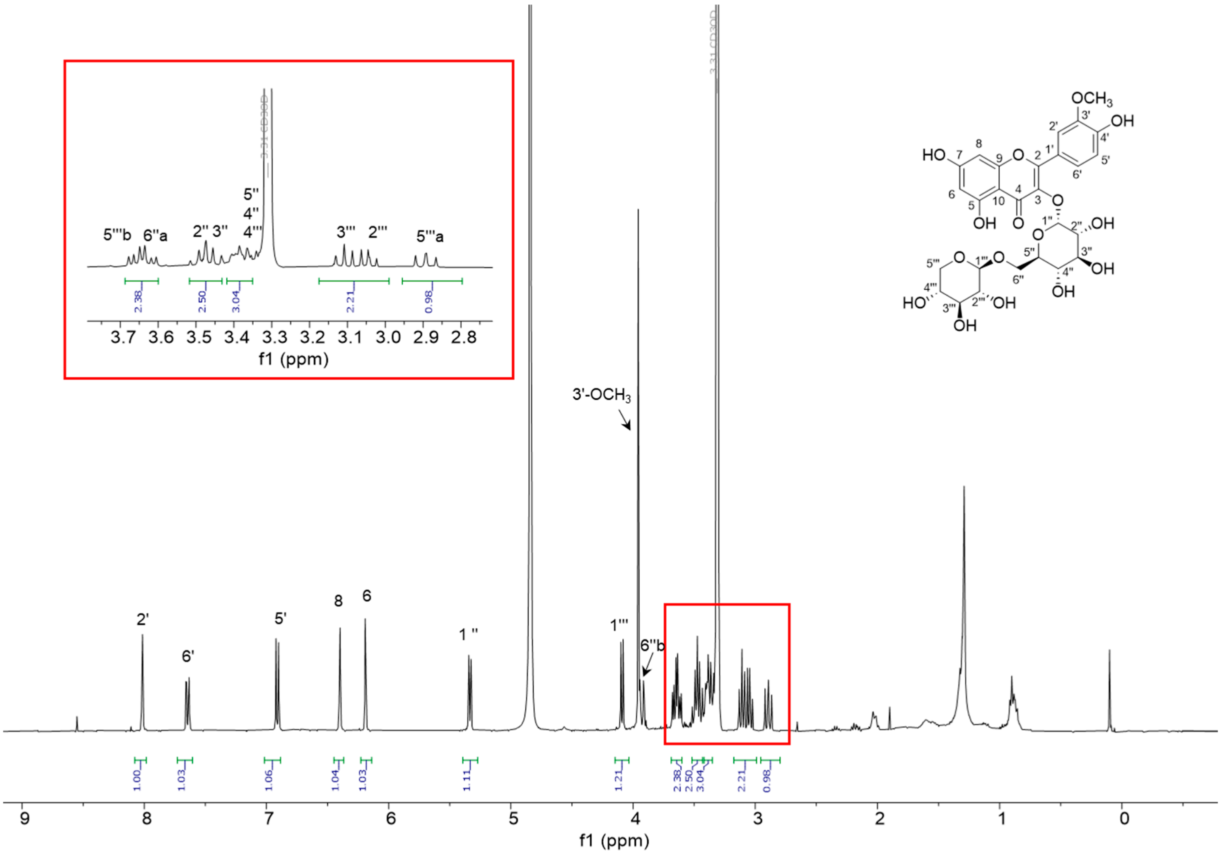

Figure S3. ^{1}H NMR spectrum (400 MHz, CD_3OD) of compound **28**

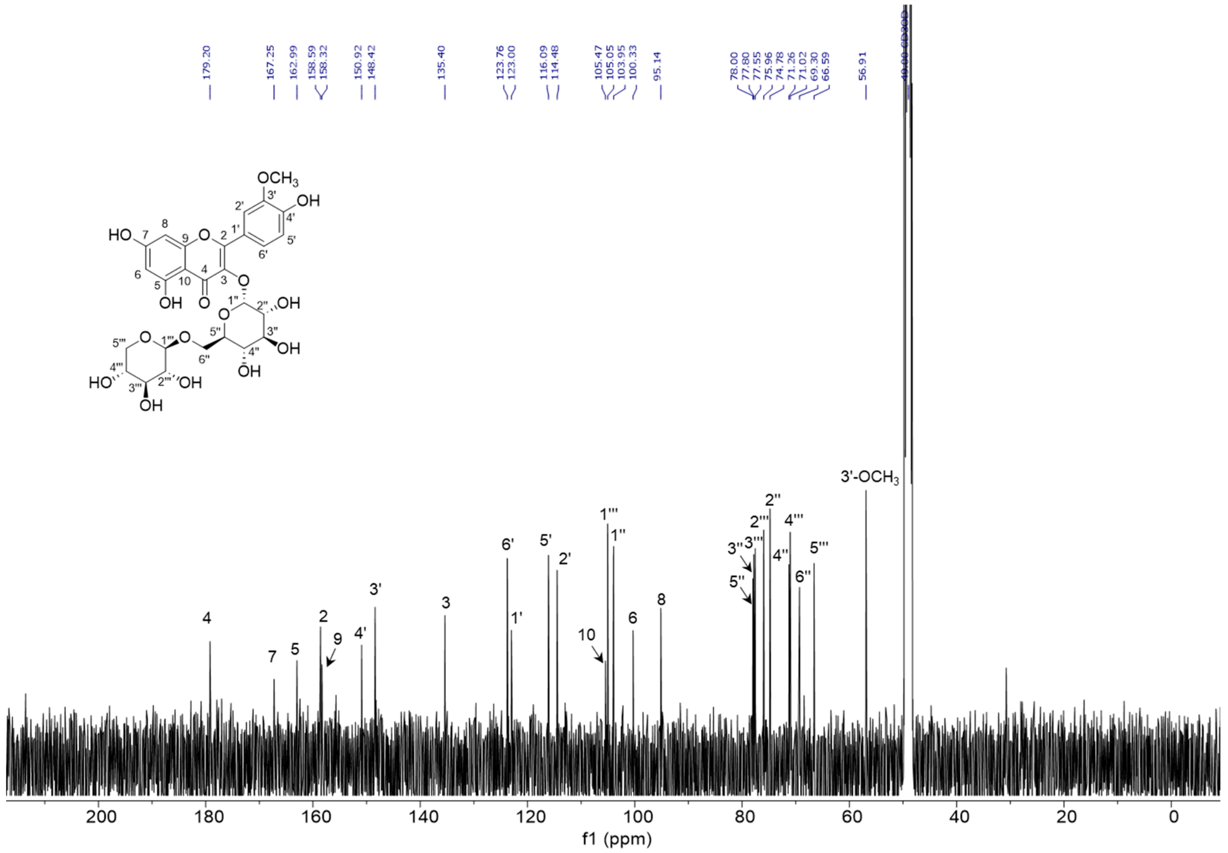

Figure S4. ^{13}C NMR spectrum (100 MHz, CD_3OD) of compound **28**

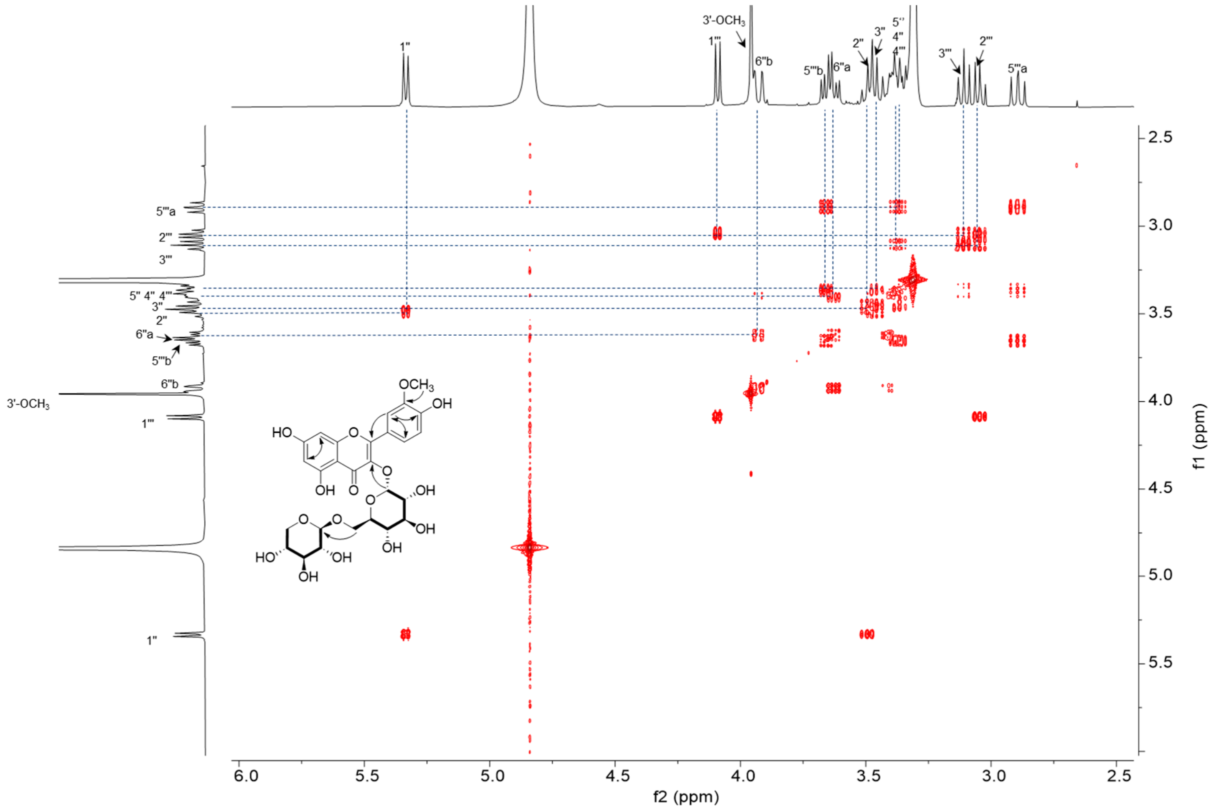

Figure S5. ^{1}H-^{1}H COSY spectrum (400MHz, CD_3OD) of compound **28**

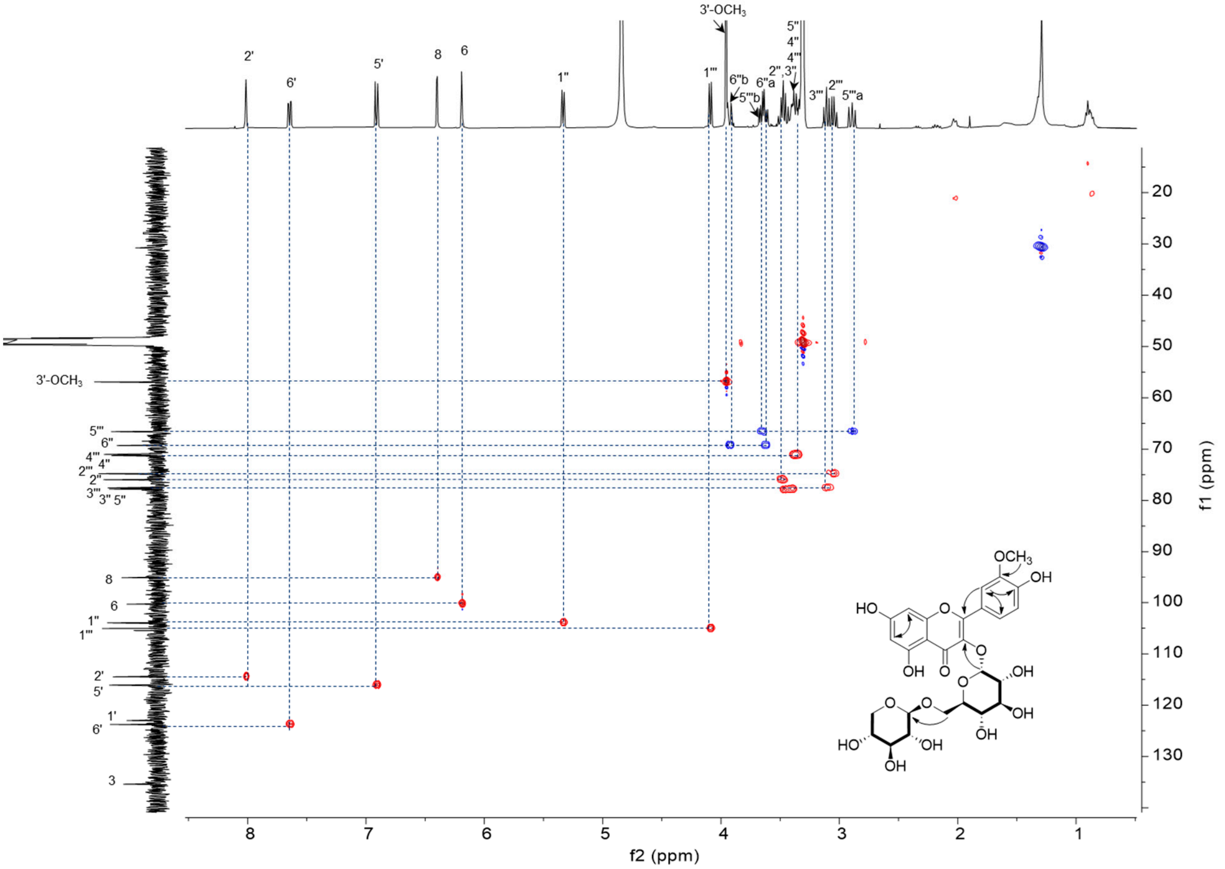

Figure S6. ^{1}H-^{13}C edited HSQC spectrum (100 MHz, CD_3OD) of compound **28**

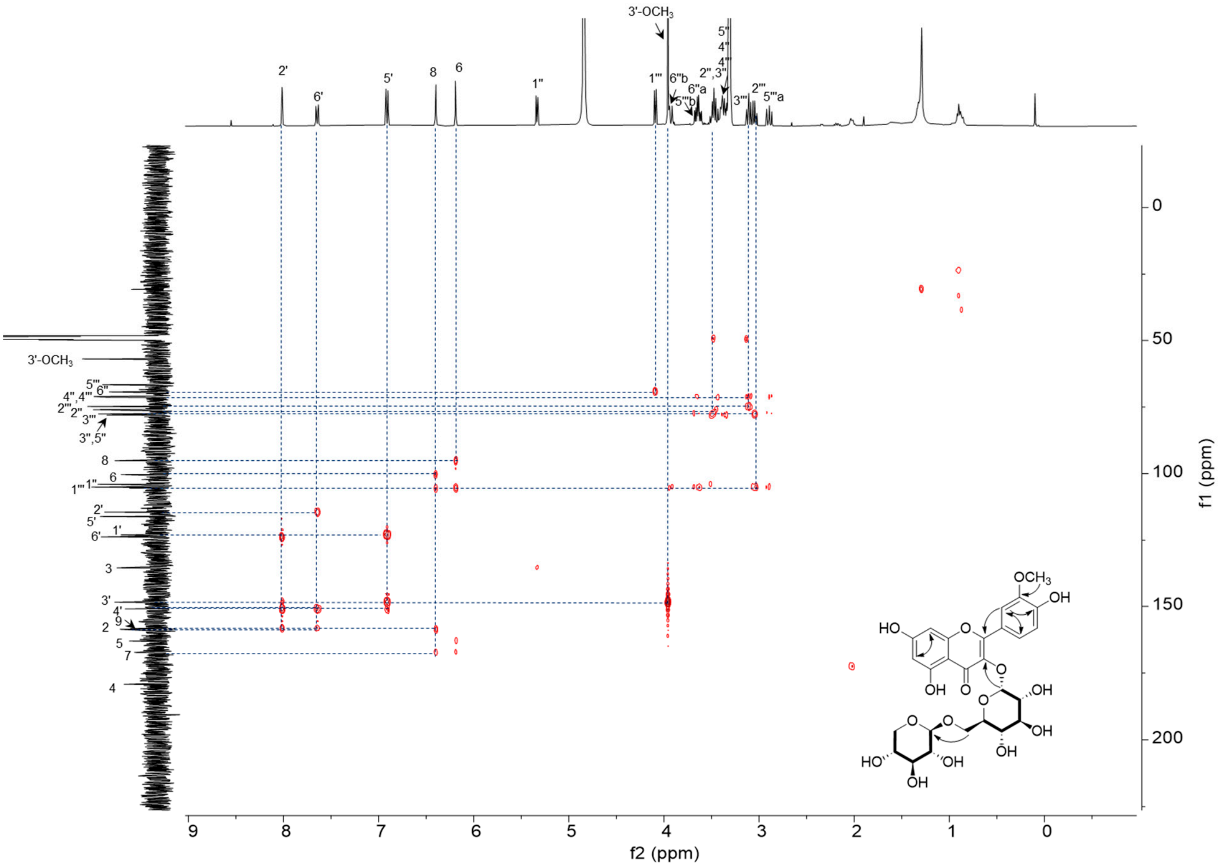

Figure S7. ^{1}H-^{13}C HMBC spectrum (100 MHz, CD_3OD) of compound **28**

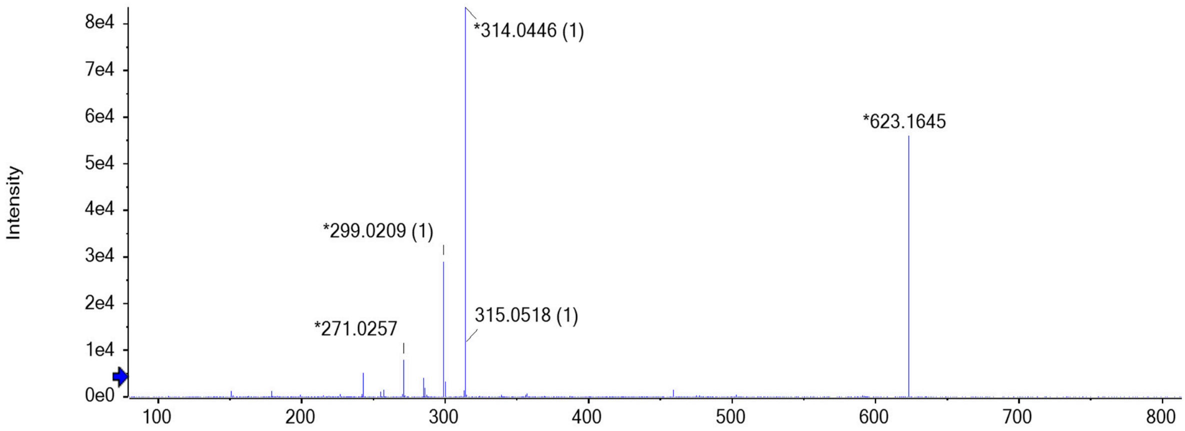

Figure S8. MS^2 spectrum of isorhamnetin 3-*O*-neohesperidoside (**A2**, compound **30**; Rt = 4.75 min; $[M-H]^-$ *m/z* 623.165)

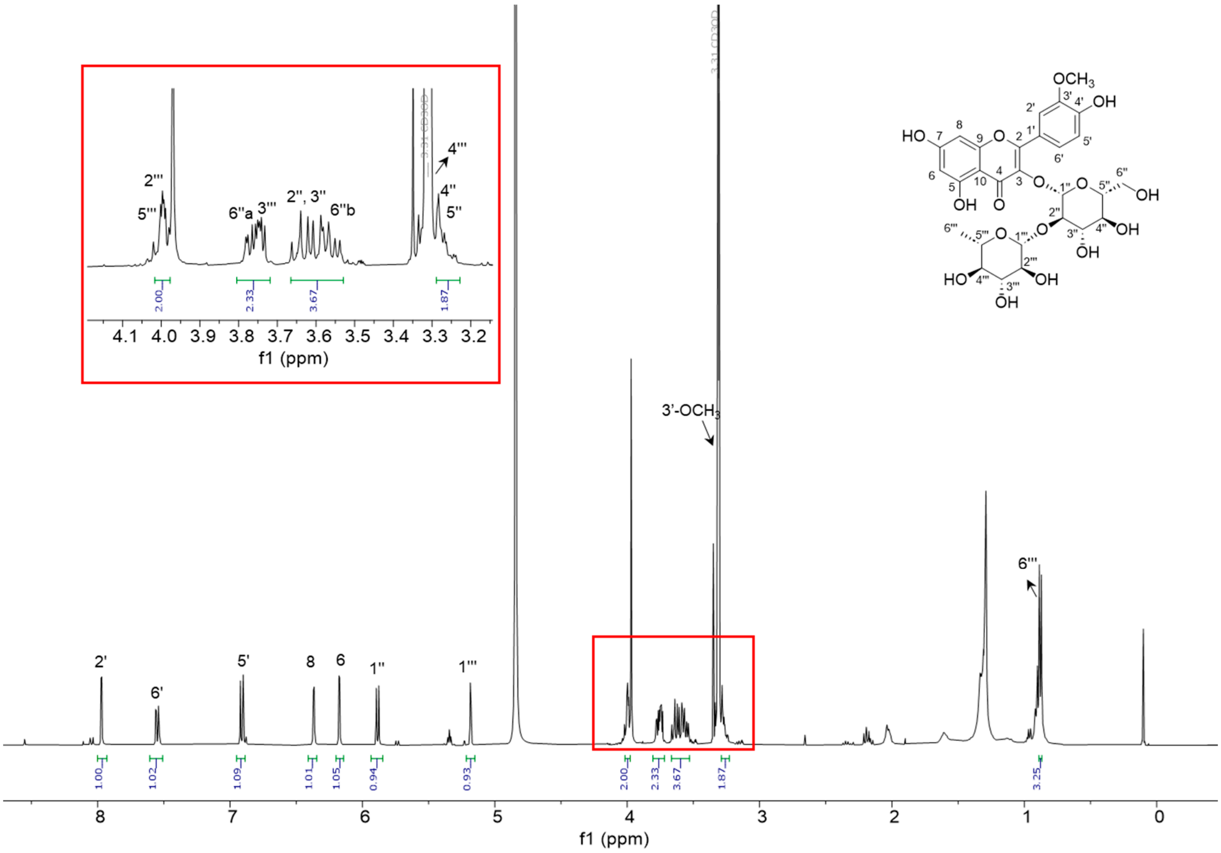

Figure S9. ^{1}H NMR spectrum (400 MHz, CD_3OD) of compound **30**

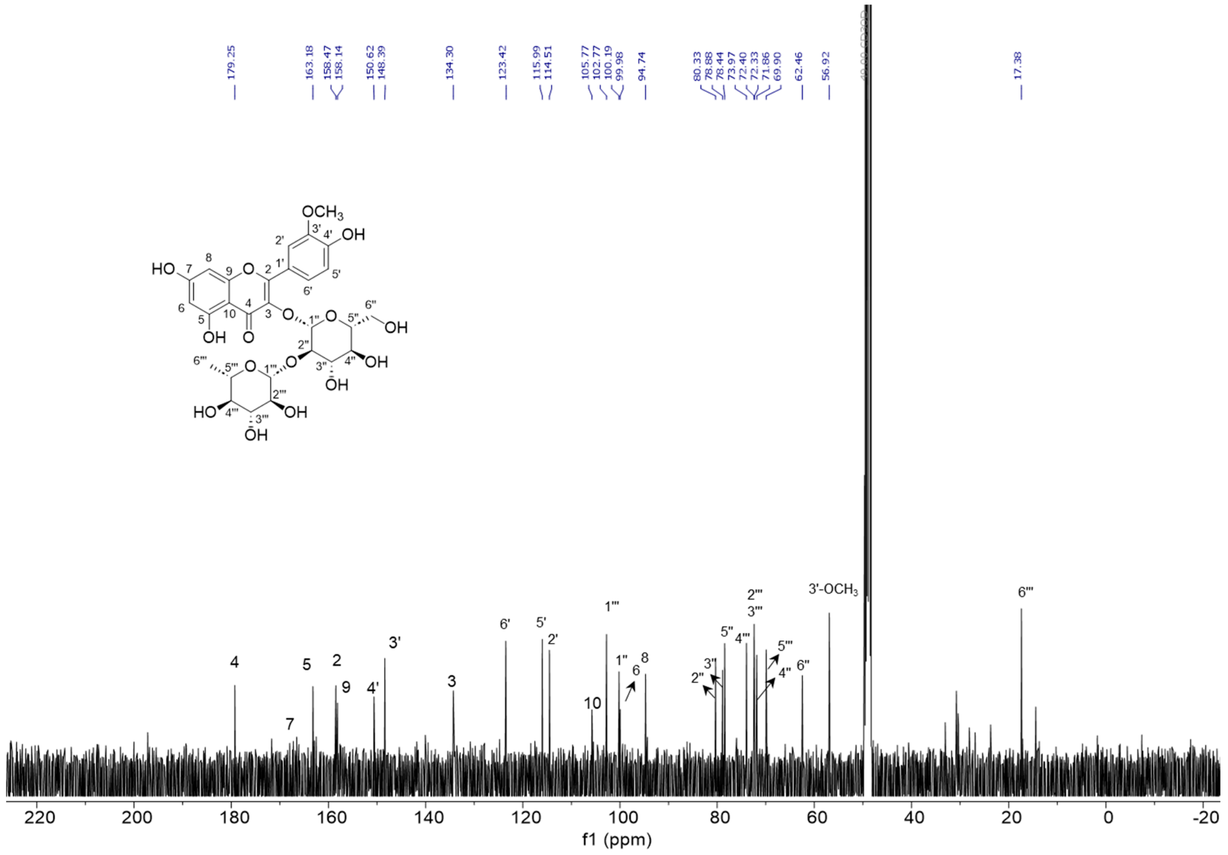

Figure S10. ^{13}C NMR spectrum (100 MHz, CD_3OD) of **30**

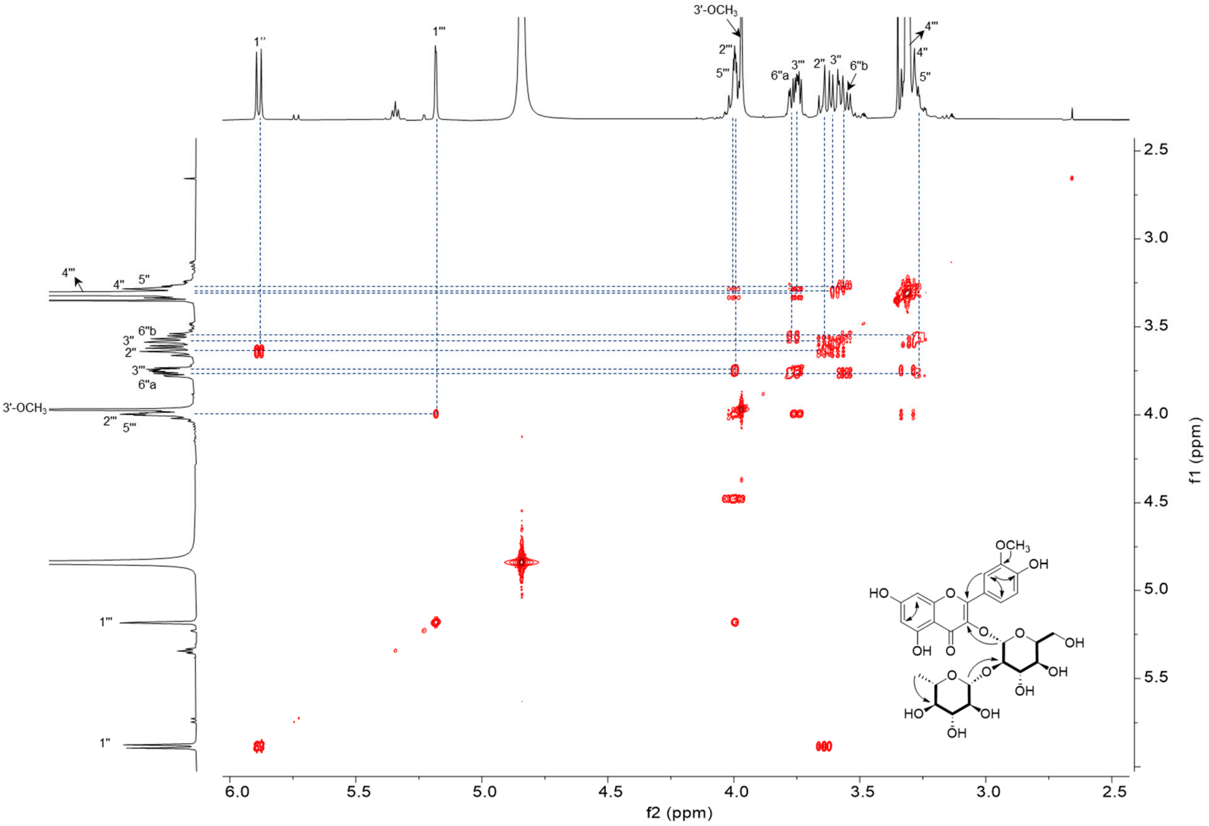

Figure S11. ^{1}H-^{1}H COSY spectrum (400 MHz, CD_3OD) of compound **30**

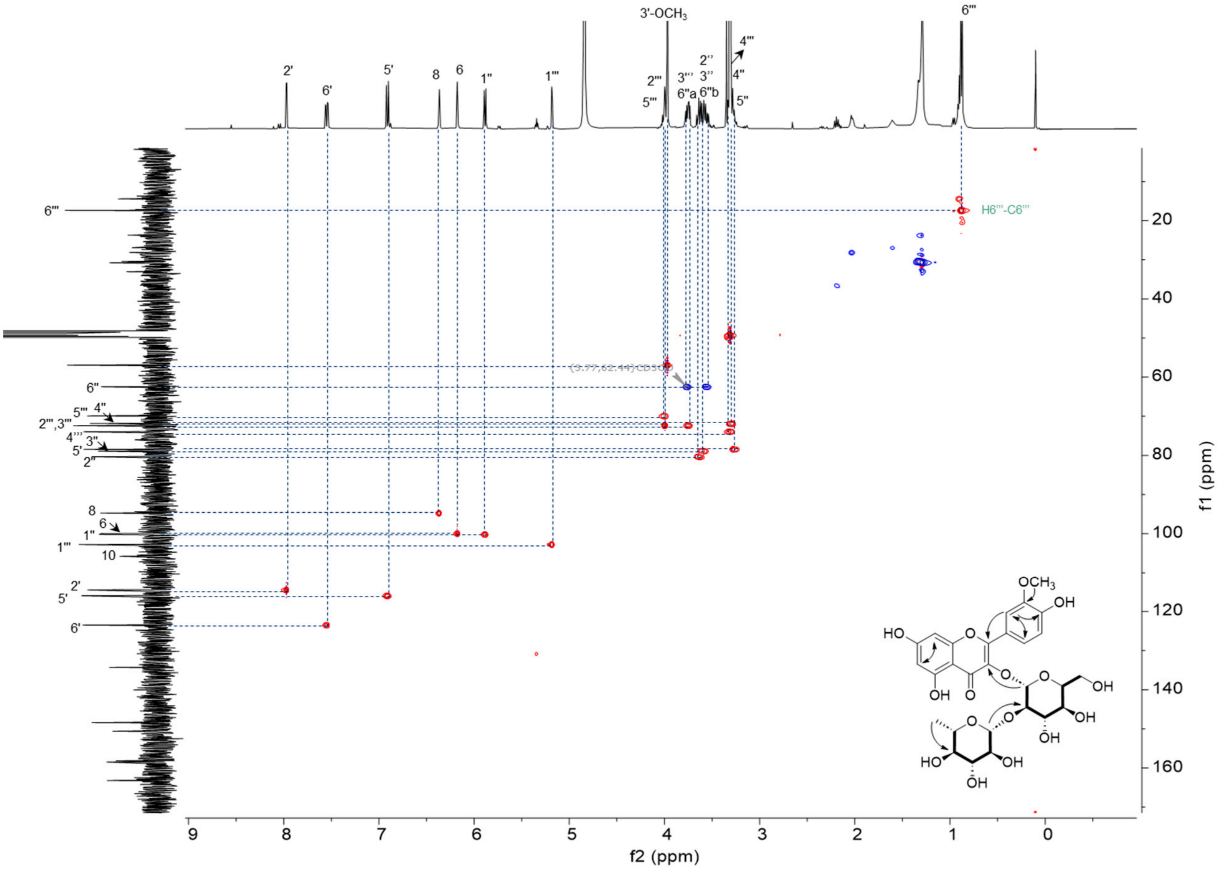

Figure S12. ^{1}H-^{13}C edited HSQC spectrum (100 MHz, CD_3OD) of compound **30**

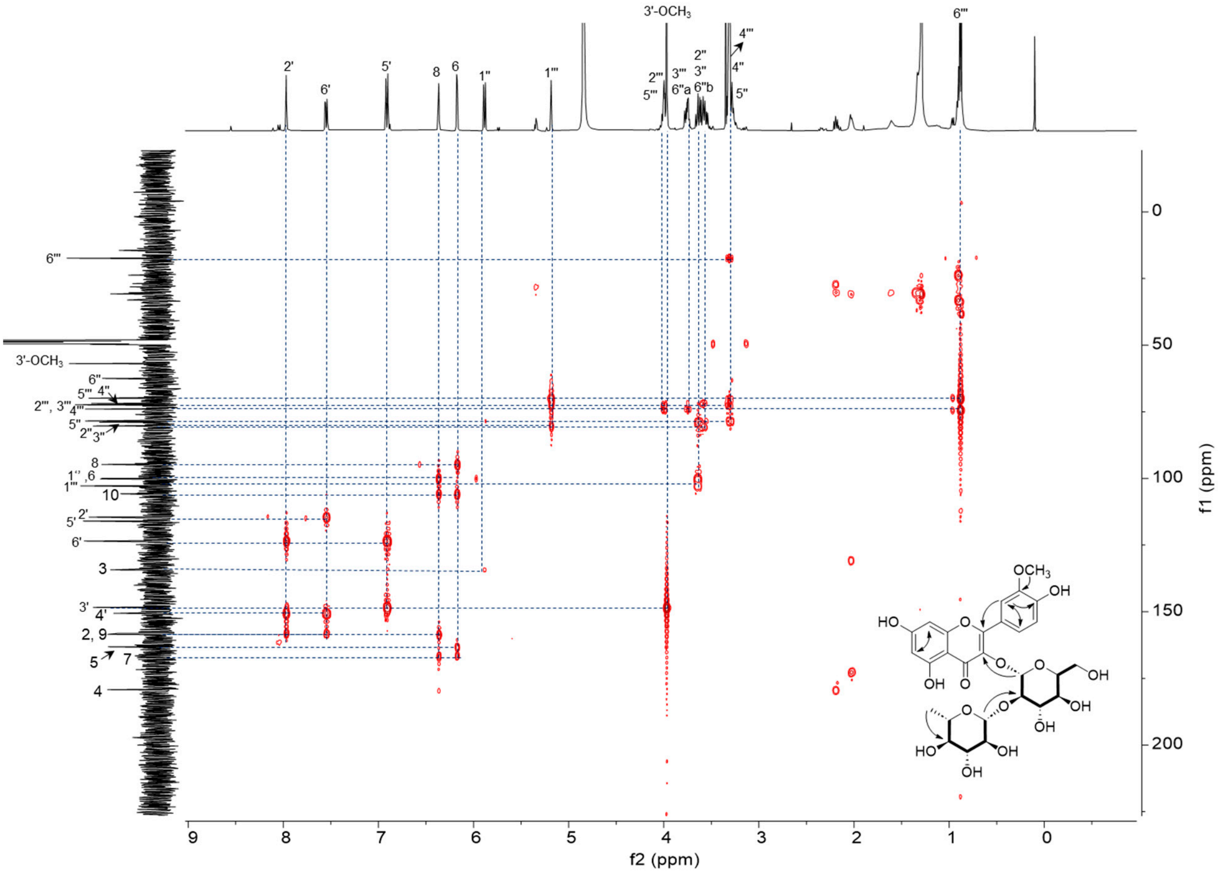

Figure S13. ^{1}H-^{13}C HMBC spectrum (100 MHz, CD_3OD) of compound **30**
